# Implementing eScreening for suicide prevention in VA post-9/11 transition programs using a stepped-wedge, mixed-method, hybrid type-II implementation trial: a study protocol

**DOI:** 10.1186/s43058-021-00142-9

**Published:** 2021-04-29

**Authors:** James O. E. Pittman, Laurie Lindamer, Niloofar Afari, Colin Depp, Miguel Villodas, Alison Hamilton, Bo Kim, Maria K. Mor, Erin Almklov, John Gault, Borsika Rabin

**Affiliations:** 1 VA Center of Excellence for Stress and Mental Health, 3350 La Jolla Village Dr, San Diego, CA USA; 2 VA San Diego Healthcare System, 3350 La Jolla Village Dr, San Diego, CA USA; 3 Department of Psychiatry, University of California San Diego, 9500 Gilman Dr, La Jolla, CA USA; 4 San Diego State University, 5500 Campanile Dr, San Diego, CA USA; 5 VA Greater Los Angeles Healthcare System, 11301 Wilshire Blvd, Los Angeles, CA USA; 6 HSR&D Center for Healthcare Organization and Implementation Research, VA Boston Healthcare System, 150 South Huntington Avenue, Boston, MA USA; 7 Department of Psychiatry, Harvard Medical School, 25 Shattuck Street, Boston, MA USA; 8 VA Pittsburgh Healthcare System, Pittsburgh, PA USA; 9 VA Department of Biostatistics, Graduate School of Public Health, University of Pittsburgh, Pittsburgh, PA USA; 10 UC San Diego Herbert Wertheim School of Public Health and Human Longevity Science, University of California San Diego, 9500 Gilman Dr, La Jolla, CA USA; 11 UC San Diego Dissemination and Implementation Science Center, University of California San Diego, 9500 Gilman Dr, La Jolla, CA USA

**Keywords:** eScreening, Implementation, Facilitation, Veterans, Suicide, PRISM, RE-AIM

## Abstract

**Background:**

Post-9/11 veterans who enroll in VA health care frequently present with suicidal ideation and/or recent suicidal behavior. Most of these veterans are not screened on their day of enrollment and their risk goes undetected. Screening for suicide risk, and associated mental health factors, can lead to early detection and referral to effective treatment, thereby decreasing suicide risk. eScreening is an innovative Gold Standard Practice with evidence to support its effectiveness and implementation potential in transition and care management (TCM) programs. We will evaluate the impact of eScreening to improve the rate and speed of suicide risk screening and referral to mental health care compared to current screening methods used by transition care managers. We will also evaluate the impact of an innovative, multicomponent implementation strategy (MCIS) on the reach, adoption, implementation, and sustained use of eScreening.

**Methods:**

This is an eight-site 4-year, stepped-wedge, mixed-method, hybrid type-II implementation trial comparing eScreening to screening as usual while also evaluating the potential impact of the MCIS focusing on external facilitation and Lean/SixSigma rapid process improvement workshops in TCM. The aims will address: 1) whether using eScreening compared to oral and/or paper-based methods in TCM programs is associated with improved rates and speed of PTSD, depression, alcohol, and suicide screening & evaluation, and increased referral to mental health treatment; 2) whether and to what degree our MCIS is feasible, acceptable, and has the potential to impact adoption, implementation, and maintenance of eScreening; and 3) how contextual factors influence the implementation of eScreening between high- and low-eScreening adopting sites. We will use a mixed methods approach guided by the RE-AIM outcomes of the Practical Robust Implementation and Sustainability Model (PRISM). Data to address Aim 1 will be collected via medical record query while data for Aims 2 and 3 will be collected from TCM staff questionnaires and qualitative interviews.

**Discussion:**

The results of this study will help identify best practices for screening in suicide prevention for Post-9/11 veterans enrolling in VA health care and will provide information on how best to implement technology-based screening into real-world clinical care programs.

**Trial registration:**

ClinicalTrials.gov: NCT04506164; date registered: August 20, 2020; retrospectively registered

Contributions to the literature

- The first, to our knowledge, to examine the impact of electronic screening on rates of mental health and suicide screening, and referral to needed treatment.
- Among the few to examine the use of an adapted Lean/SixSigma Rapid Process Improvement Workshop as part of a facilitated multi-component implementation strategy.
- Combines an effectiveness trial of an innovative screening technology with a novel implementation strategy that will be evaluated with state-of-the-art implementation science methods in a hybrid type II study.

## Background

Veterans disproportionately account for all known suicides in the USA, accounting for 20–22% of those who end their lives [1, 2]. After 9/11, suicide rates became more frequent in veterans than civilians [3, 4], a surge that has been called an epidemic [5, 6]. Veterans diagnosed with mental health disorders, including posttraumatic stress disorder (PTSD), depressive disorders, and alcohol problems have increased risk for suicide [7–14], and over 90% of suicide victims have a diagnosable mental health and/or substance use disorder [15]. The strong association between suicide and mental health conditions presents opportunities for suicide prevention. First, systematic screening can improve the detection of mental health problems commonly associated with suicide [16–18] and can facilitate connection to mental health treatment [19]. Effective mental health treatment can reduce suicide risk and lower suicide rates [15]. Second, early identification of the mental health and substance use conditions can immediately identify those who should be targeted for comprehensive suicide risk assessment and intervention.

Of importance, screening for suicide risk and other mental health conditions related to suicide risk at the first contact with a healthcare organization is considered vital to enhancing access to appropriate care and is a best practice in the National Zero Suicide Framework [20]. One such venue for screening at first contact is the Veterans Health Administration (VHA) Transition Care Management (TCM) programs that coordinate health care for Post-9/11 veterans at the point of enrollment, before establishing primary care. The current screening and documentation processes rely on clinical staff to collect information from veterans verbally or with paper forms resulting in inefficiencies that can result in delays for further assessment, referrals, and/or treatment. Previous research showed that about half of the veterans who present for the first time in VHA with recent suicidal thoughts do not receive comprehensive suicide risk evaluation possibly due to the delay in entering data or lost paper screens [15]. A technology-based solution could expedite the assessment and treatment of veterans.

### Electronic screening

Electronic self-report screening (ESRS) can be an effective assessment tool for timely detection and intervention of suicidal ideation and other mental health symptoms [21, 22]. There is high reliability between electronic and paper-screening [23], and individuals often prefer electronic screening over the human interview for sensitive subject matters such as substance abuse or suicidal ideation [22]. ESRS provides patients with prompt access to their results, encourages patient-provider communication, and aides in follow-up care [15, 21, 22]. In addition, ESRS can result in time savings, fewer organizational resources, flexibility of collection location, and reduction of error and biases [21–24].

eScreening is a web-based, patient-facing ESRS system developed with user-centered design methods from Veteran and staff user feedback, and refined to improve the quality, documentation, and access to care [25]. It can read and write to the VHA electronic medical record (EMR) system enabling secure real-time alerts to clinicians for evaluating and triaging, generating aggregate clinic data for managers, and providing personalized feedback for veterans. Across multiple VHA facilities, eScreening has been utilized over 34,000 times with veterans in TCM, primary care, and mental health settings in the provision of screening and measurement-based deployment of evidence-based psychotherapy (EBP). In 2016, the eScreening program was named a Gold Standard Practice for diffusion throughout VHA by the Under-Secretary for Health [26].

### Implementation strategies

There is widespread agreement about the importance and potential benefits of health technology, yet difficulties in understanding how best to implement health technologies have slowed progress in this area [27]. Key strategies for a successful implementation of health technology include planning, training and assessment of staff, and continuous evaluation and monitoring [27]. Other factors identified were related to the characteristics of the intervention (e.g., its cost, complexity, and adaptability), the characteristics of the staff, and support for the digital interventions [26].

A wide range of quality improvement methods has been used to support the implementation of interventions and processes in healthcare, including the Lean/Six Sigma Rapid Process Improvement Workshop (RPIW) [28, 29]. Multiple healthcare institutions have improved the quality of care through the utilization of variations of RPIWs [30, 31]. RPIWs can be effective for implementing evidence-based practices in behavioral health care [32], and they have the advantages of being customer/patient-centered and balancing the role of both measurement/data and people in effectively implementing an EBP [28].

Implementation facilitation, another strategy that has been used broadly in the VHA. It involves a process of interactive problem-solving and support that occurs in the context of a recognized need for improvement and within a supportive interpersonal relationship [33, 34] to implement a new intervention or practice. Implementation facilitation provides a mechanism to address factors that impede the uptake of an innovation regardless of the source of difficulty (e.g., characteristics of the people, intervention, or the context) [35]. A systematic review showed that primary care settings receiving facilitation were more likely to adopt evidence-based guidelines [36], and several studies conducted in the VHA have shown that facilitation improves implementation of complex evidence-based programs, including an outreach program for veterans with serious mental illness and Primary Care-Mental Health Integration treatment services for veterans with dual diagnoses [37–39]. External facilitation, which leverages the process knowledge and subject matter skills and expertise of an external (outside the site) facilitator to work with an internal (within the site) facilitator, is a powerful strategy to improve implementation [40]. External facilitation has the potential to overcome many existing barriers to health care research by strengthening relationships between researchers and stakeholders and accelerating the implementation of innovative care practices [39].

Despite some evidence that electronic screening may be effective for timely detection of, and intervention for, suicidal ideation and other mental health symptoms, additional effectiveness and implementation research is warranted to evaluate the impact of eScreening within VHA. The dual goals of this trial are to address questions of the impact of eScreening compared to screening as usual and evaluate a multicomponent implementation strategy (MCIS) that involves training, RPIW, and external facilitation. We will also assess how contextual factors influence the implementation of eScreening between high- and low-eScreening adopting sites.

### Implementation framework

**Fig. 1:**
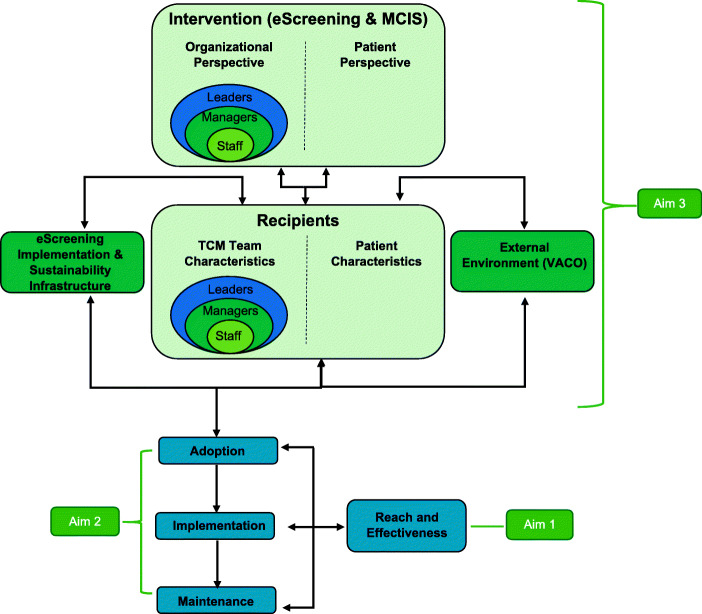
Practical Robust Implementation and Sustainability Model adapted for eScreening

We will use the Practical Robust Implementation and Sustainability Model (PRISM; [41]) to guide the implementation and evaluation. PRISM is a contextually extended version of the more broadly known RE-AIM (Reach, Effectiveness, Adoption, Implementation, Maintenance) framework [42, 43]. The PRISM contextual factors include organizational and patient perspectives of the intervention, characteristics of the recipients, the infrastructure to support implementation and sustainment, and external environment. These inter-relational contextual factors influence each other and the RE-AIM outcomes (See Fig. 1).

We selected PRISM because of its ability to identify contextual factors that are hypothesized to influence RE-AIM implementation outcomes. PRISM integrates contextual factors with the RE-AIM outcomes in a single model designed to be practical and actionable for practitioners and researchers to guide implementation [44–47]. Important elements to improve program implementation based on PRISM include addressing barriers of front-line staff, training, leadership support, observing results, and adjusting processes accordingly, as well as ensuring the adaptability of protocols that fit the multi-level context. Moreover, PRISM’s relative intuitiveness and emphasis on the alignment or fit among context, implementation strategy, and outcomes are important to implementation and sustainability success. PRISM has been used to guide the implementation and evaluation of programs in the VHA with great success [48].

### Aims

The specific aims of the study are as follows:

Aim 1: Evaluate eScreening, compared to paper and verbal screening (treatment as usual; TAU), guided by the RE-AIM outcomes of PRISM in 8 TCM programs, using a cluster-randomized, stepped-wedge design. Hypothesis 1 (Reach): Compared to TAU, eScreening will result in a significantly higher proportion of veterans being screened. Hypothesis 2 (Effectiveness): 2a: Compared to TAU, eScreening will result in significantly less time from enrollment to mental health and suicide screening. 2b: Compared to TAU, eScreening will result in a significantly higher proportion of veterans being referred to needed care.

Aim 2: Evaluate the feasibility, acceptability, and potential impact of the MCIS, guided by the Adoption, Implementation, and Maintenance RE-AIM outcomes of PRISM, using mixed methods.

Aim 3: Describe and compare high- and low-eScreening reach sites guided by contextual constructs of PRISM using qualitative comparative analysis to explore factors influencing the reach of eScreening and the use of the MCIS.

## Methods

### Design

**Fig. 2:**
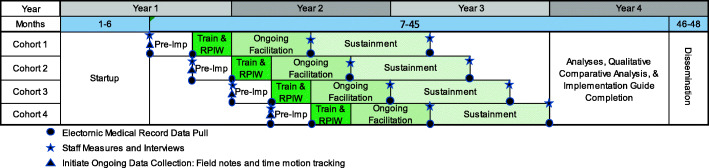
Project overview (*N*=8 sites; 45 staff; 27,600 Veterans)

This paper follows the Standards for Reporting Implementation (StaRI; [49]) Studies and the Standard Protocol Items: Recommendations for Interventional Trials (SPIRIT [50];) checklist to describe this stepped-wedge (SW), mixed-method, hybrid type-II implementation trial of eScreening in TCM programs in 8 VHA sites. Sites will be stratified by size and block randomized into four-step/crossover cohorts. Figure 2 presents an overview of the project across the 4-year trial.

### Sites and participants

Each VA Healthcare System has a TCM program that screens newly enrolling post-9/11 Veterans for care management and referral to specialty care. Programs vary based on the needs of the system and range in size from 1 to over 20 staff and average 30–375 newly enrolling veterans per month. Veterans seen in these programs have a median age of 35 and over 30% are women [51, 52]. They are the most diverse group of veterans with 20.5% non-White and 12% Hispanic [49, 50]. Post-9/11 veterans also have higher levels of educational attainment as compared to those from other eras with 32% of post-9/11 veterans holding a bachelor’s degree or higher [49, 50]. In collaboration with the national TCM program office, we identified 8 VA Healthcare Systems that are interested in deploying eScreening. We chose sites based on the level of interest from their field and facility leadership (see Table 1). We also attempted to identify sites with diverse TCM programs based on rurality, TCM staffing level, and patient volume. Based on our preliminary work, we anticipate a sample of 45 TCM and related staff will be enrolled in this study. Patient-level data will be collected from EMR, and no veterans will directly participate; their involvement will only be through receiving services from the TCM program at our study sites.

**Table 1:** Study implementation sites

Site	Region	Rurality	# of TCM staff	Veterans/month
1	MidWest	Urban	6	150
2	West	Rural	5	30
3	Southeast	Urban	8	375
4	Southeast	Small City	4	30
5	Southwest	Urban	8	300
6	Southwest	Rural	4	45
7	West	Urban	7	130
8	MidWest	Rural	6	90

*TCM* Transition Care Management

### Procedures

Internal facilitators, TCM staff and eScreening implementation-related stakeholder staff at each site will be invited to participate in the study. An informational session about the study’s leadership and purpose, selection of participants, and use of data will be conducted. Following this informational session, research staff will consent interested participants. After consent has been signed, enrolled staff members will receive a link to an online survey and will be scheduled for a preliminary interview by the evaluation lead. If staff turnover occurs, we will attempt to assess the staff member prior to leaving and replace and train another participant with similar functions within the clinic. These recruitment procedures will be reviewed and adjusted, if needed, in the startup period. Each of the four-step/crossover cohorts will go through the following phases sequentially during the study.

### Pre-implementation phase

This phase will last 3 months during which the external facilitator team will work with the internal facilitators to gather pre-implementation information including detailed information on the processes in place for TCM screening upon enrollment, identify points of contact for local IT, establish communication with TCM staff and others working with the TCM staff, recruit staff participants for the study, and begin ongoing tracking of process data from field notes and time-motion tracking. TCM staff names, clinic names, note titles used in the EMR, and clinical screens completed by program staff will be gathered and used for subsequent development of eScreening user accounts and content customization during the implementation phase. The implementation team will also provide psychoeducation to the staff on the importance of screening. This phase will serve as an attention control condition to which the baseline control and intervention conditions will be compared.

During pre-implementation, TCM teams will continue usual screening procedures that involve interview or self-report, paper-based collection of post-9/11 screening measures, including the system-wide standardized assessments of depression, PTSD, and alcohol use (PHQ-2, PCPTSD, and Audit-C, respectively). The Nationally VHA-mandated process is for veterans who score positive on the PHQ-2, PCPTSD to then be administered the Columbia Suicide Severity Rating Scale (C-SSRS), which collects more information regarding the risk of suicide. Veterans who are positive on C-SSRS then receive a Comprehensive Suicide Risk Evaluation (CSRE) and are referred for appropriate follow-up. A detailed description and flow map of the current screening process at each site will be developed by the external facilitator (research team) and internal facilitator (site staff) with information from the TCM staff prior to the implementation phase.

### Active implementation

The MCIS was developed over the past 7 years and consists of (1) eScreening software provision, (2) training, (3) RPIW, and (4) ongoing external facilitation. We developed our MCIS to address specific eScreening implementation barriers that we found in our prior research [53]. In order to address system-level barriers related to VA Office of Information Technology (IT) support, we developed a technical support infrastructure for eScreening using existing VA IT resources as part of eScreening provision. The training component addresses educational barriers regarding eScreening use and the available research evidence for electronic screening. External facilitation also addressed educational barriers as well as technology-related and other unforeseen challenges. The RPIW process will address leadership support, staff buy-in, and resources needed. All stakeholders will participate in the process to develop a site-specific plan for implementation. The RPIW specifically includes a section where the team generates possible barriers and solutions.

#### Training

eScreening training will be virtual and asynchronous and include a 1-h instructional PowerPoint that will be presented by the research team. The PowerPoint is followed by an hour of tutorial videos that demonstrate the key steps involved in using eScreening. Hands-on training for users will be available in group format or individually by the training staff as requested by the TCM site staff. Additional training materials can be accessed via a shared site that includes a series of quick guides to address eScreening customization, assessment set-up, and dashboard use. Technical information and training materials also will be available on the shared site, to include frequently asked questions, tutorial videos, a technical manual and user guide, a large amount of training information, and the eScreening playbook. We will update these materials to include the most recent information on eScreening and to support the training protocol.

#### RPIW

The 3-day RPIW will be facilitated virtually by the external facilitator with assistance from the onsite internal facilitator and will include the TCM team, related staff (i.e., medical support staff, clerks), and other site stakeholders. The first day of the workshop includes training participants in the Lean/SixSigma principles and introduces a summary of the information gathered in the Pre-Implementation Phase, including a graphic of the current state screening process map that will then be refined and finalized. The second day consists of collective efforts to map a targeted future state, conduct a gap analysis, and identifying relevant factors and barriers unique to the site. The third day is dedicated to the repetition of action planning, execution, and reevaluation to finalize the screening target state and identify clinically meaningful goals for improvement. Using a Plan-Do-Study-Act (PDSA) framework [54], the plans to achieve the target state are enacted with a detailed plan that includes who, what, and when for each step in the plan. Due to the flexibility of eScreening and the implementation strategy, each TCM program may choose to integrate eScreening into their workflow based on the specific needs of their program, available resources, and other factors.

#### External facilitation

External facilitation will include a primary external facilitator from the eScreening team who will work with the site internal facilitator to schedule meetings, training sessions, and phone calls. The external facilitator will be the main point of contact for implementation-related questions. The internal facilitator, selected during the startup period, will work with the external facilitator to navigate internal site systems (i.e., local leadership, IT, logistics) and serve as a champion for the eScreening project at each site.

### Data sources and analytic approach

We use a mixed-methods design and will collect a combination of quantitative and qualitative data from multiple stakeholders and at multiple time points. Below, we describe the data collection and analysis for each study aim.

#### Aim 1

We will collect data in order to evaluate eScreening, compared to TAU, guided by the RE-AIM outcomes of PRISM in 8 TCM programs, using a stepped-wedge design with cluster randomization. These data include the number of veterans that enrolled in the healthcare system and the date and time they enrolled; the date and time that they received PHQ-2, PCPTSD, and Audit-C, CSSRS, and the disposition (positive/negative screen); date and time they received a CSRE; and the number of mental health care referrals (see Table 2). These data will be used to calculate the overall rate of screening completion and referral to mental health care during the baseline control period and the average length of time to screening completion. This will be repeated at post-implementation and sustainment. Based on the average enrollment data for our sites over the past year (expected 144 veterans average per month across sites), approximately 27,600 veterans will enroll in VA healthcare during the 27-month baseline control, pre-implementation, implementation, and sustainment time periods.

**Table 2:** Aim 1 eScreening Data Elements guided by PRISM

RE-AIM Dimension	Measures	Data Source(s)	Data Type	Level
Reach Proportion of Newly Enrolling post-9/11 veterans who received screening	• % of eligible veterans who were screened & unscreened (eScreening versus Paper/Oral Screening)	• EMR Query • eScreening Query	• Quant	• Patient
Effectiveness The impact of eScreening on important outcomes: speed of screening completion and rate of referral to needed care	• Enrollment date/time	• EMR Query	• Quant	• Patient
	• Mental health (PHQ-2, PCPTSD, Audit-C) and suicide risk screening (CSSRS, CSRE) date/time and score	• EMR Query	• Quant	• Patient
	• % Consults submitted to Mental Health from TCM clinics with eScreening vs. paper/ oral screening	• EMR Query	• Quant	• Patient

*Quant* Quantitative, *EMR* Electronic Medical Record

We powered the study for the intervention effect of the effectiveness outcomes in Aim 1. We assumed a common intervention effect across all cohorts/steps and Hierarchical Linear Modeling (HLM) [55] to account for clustering, including a fixed effect for cohort/step of crossover to account for secular trends and an indicator of the intervention phase change (e.g., control vs. intervention) to provide intervention effects. Power was calculated based on established methods for stepped wedge trials [56]. We set type I error rate alpha = 0.05, Cohen’s *d* (or *h* for binary outcomes) effect size = 0.1, power = 0.8, and assumed an intraclass correlation (ICC) = 0.20, which is a conservative estimate based on similar studies [57, 58]. Under these assumptions, the estimated sample size needed for the proposed study is approximately *N* = 5000 participants. Data from our pilot study show effect sizes that are all above this detectable effect size [15]. Given that 144 new post-9/11 veterans are enrolled on average across implementation sites each month and we will collect data at each site over a 24-month period, the study is sufficiently powered to detect effect sizes observed in similar studies.

HLM will be used as the primary statistical model due to the nested (or clustered) structure of the data (veterans [level 1] nested within TCM clinics [level 2] nested within implementation site [level 3]), with random assignment occurring at the implementation site level. Demographic information about participants during the MCIS and control phases will be statistically compared within and between sites to ensure comparability. Any characteristics that differ between the intervention and control groups will be included as covariates in subsequent models to minimize bias. Fixed effects will be included in each model to account for the study phase (i.e., baseline control, pre-implementation/attention control, MCIS, and sustainment) and step/crossover cohort, to account for secular trends. We also will be able to test interactions between the study phase and step to determine whether intervention effects differed by cohort and whether intervention effects varied between TCM clinics within implementation sites. Separate models will be tested to determine whether a greater proportion of veterans were screened for mental health and suicide risk (Reach) and referred to care (Effectiveness) during the MCIS and/or sustainment phases relative to the baseline and/or attention control phases. Additional models will test whether the mean number of days between enrollment and screening were lower (effectiveness) during the MCIS and/or sustainment phases relative to the baseline and attention control phases. In all analyses, we will set statistical significance at alpha = 0.05 and use Holm-Bonferroni adjustments for ≤5 tests [59] and false discovery rate methods for > 6 tests [60]. When multiple correlated outcomes (dependent outcomes) are analyzed with each hypothesis, corrections will be calculated based on the effective number of independent tests when applying the multiple comparison procedures [61]. Missing data are expected to be limited and are readily incorporated in HLM if the data can be assumed to be missing at random using maximum likelihood estimation [62]. If the data are determined not to be missing at random, missing data mechanisms will be built into the target statistical models.

#### Aim 2

To evaluate the feasibility, acceptability, and potential impact of the MCIS, we will use a mixed methods approach and collect both quantitative and qualitative data guided by the RE-AIM outcomes of PRISM. Table 3 summarizes the data to be collected based on the selected RE-AIM dimensions, adoption, implementation, and maintenance. For the replication cost, we will use a time tracker previously used for VA implementation efforts [63]. The tool will be customized for this study and used to incrementally capture all facilitation activity by the external facilitator. We will use an estimated time spent on implementation by the internal facilitator and other site staff based on the percentage of their work hours officially dedicated to implementation. Time spend on implementation will then be quantified and used to develop a replication cost estimate by site.

**Table 3:** Aim 2 MCIS Data Elements guided by RE-AIM outcomes of PRISM

RE-AIM Dimension	Proposed Measures	Data Source(s)	Data Type	Level
Adoption Absolute number, proportion of clinics & providers who are willing to *initiate a program* compared to eligible non-participants	• % of TCM Clinic staff that attend the eScreening training and RPIW	• RPIW Invitation • Attendance count	• Quant	• Provider & Clinic
	• Barriers and facilitators to adoption	• Observation • Staff Survey • Staff Interview	• Qual • Quant • Qual	• Clinic
	• Strategies used to increase adoption (including non-TCM interest)	• Observation • Staff Interview	• Qual	• Clinic
	• Reasons for or against adoption	• Staff Interview	• Qual	• Provider & Clinic
Implementation The intervention agents' fidelity to elements of an intervention's protocol (includes consistency of delivery as intended), adaptations to the intervention, & replication cost	• % of TCM Clinics & providers that use eScreening for screening	• eScreening Query	• Quan	• Provider
	• Documented adaptations to eScreening or the MCIS (including non-TCM implementation)	• Adaptation tracking log	• Qual	• Clinic
	• Barriers & facilitators to implementation	• Observation • Staff Interview	• Qual	• Clinic
	• Acceptability and feasibility of eScreening and MCIS	• Staff Survey • Staff Interview	• Quant • Qual	• Provider & Clinic
	• Implementation agents (internal external facilitators) time (cost)	• Time tracker	• Quant	• Clinic
Maintenance Extent to which eScreening becomes institutionalized or part of the routine practices	• % of TCM clinics and providers that use eScreening 9 months post-MCIS	• eScreening Query	• Quant	• Provider & Clinic
	• Barriers & facilitators to sustained use	• Staff Interview	• Qual	• Clinic

*Quant* Quantitative, *Qual* Qualitative, *RPIW* Rapid Process Improvement Workshop, *TCM* Transition Care Management, *MCIS* Multicomponent Implementation Strategy

We will use descriptive statistics to summarize quantitative measures for each PRISM outcome using 50% as a benchmark for success. Adoption will be calculated as the overall number and proportion of TCM clinics that are willing to initiate eScreening, relative to the total number of TCM clinics across implementation sites and within each implementation site, as well as the overall number and proportion of providers who are willing to adopt eScreening relative to the total number of providers across implementation sites, across TCM clinics at each implementation site, and within each TCM clinic. Implementation will be calculated as the proportion of TCM clinics and providers within clinics who implement eScreening. We use the scales developed by Weiner et al. [64] to calculate mean ratings of acceptability and feasibility of the MCIS across providers within TCM clinics and across implementation sites. Time tracker data will be analyzed using the VA general ledger, which includes all labor costs including employee benefits and employer contributions to taxes. Indirect costs should be incurred in proportion to direct costs and will be estimated based on VA Health Economics Resource Center (HERC) guidance [65]. Maintenance will be calculated as the proportion of TCM clinics and providers within clinics who implement eScreening during the sustainment phase (i.e., the 9-month period following initial implementation).

An experienced member of the research team will conduct semi-structured interviews. Interviews will be audio-recorded, transcribed, cleaned, and entered into ATLAS.ti [66]. This sequential process of data collection will allow us to both identify emergent themes throughout the data collection process and to triangulate already collected data. A key aspect to this analysis is to answer these questions: What influences the adoption of eScreening by providers? What factors influenced the implementation of eScreening? What factors promote maintenance? The analysis will also answer the bigger question of why providers do or do not implement eScreening, including understanding any practical clinic workflow reasons for use or non-use, or key underlying characteristics of eScreening program or provider. The analysis will consider emergent themes using an editing approach [67].

Adaptations to eScreening, the MCIS, and study processes will be documented throughout the study period (i.e., pre-implementation, implementation, sustainment) using a real-time tracking system that has been developed and used in prior research studies in the VA [68]. A member of the research team will add adaptations to this system weekly and solicit adaptations from the site champions during regular meetings. In addition to adaptations, periodic reflections on the study process will also be documented to provide contextual richness to the data [69].

Using a mixed-methods convergent design approach [70, 71], the qualitative research core team will analyze the data concurrently with the quantitative data to explain and support/refute the quantitative data and add to insights regarding future implementation research and dissemination efforts. Table 4 shows how these two types of data sources will be integrated to answer this study’s questions.

**Table 4:** Aim 2 Convergent Mixed Method Analysis using PRISM outcomes

Aim 2 PRISM Outcome	
Adoption	Goal: Characterize what influences eScreening adoption (50% benchmark) • Quant data: % of TCM Clinics & providers • Qual data: Strategies used, reasons to adopt or not from observations and interviews
Implementation	Goals: Characterize implementation processes of sites, feasibility, acceptability, calculate replication costs • Quant data: % of clinics and providers implement eScreening (50% benchmark), time tracker, and acceptability and feasibility questionnaires • Qual data: Adaptations to intervention and implementation, barriers and facilitators to implementation, acceptability and feasibility of MCIS from observations and interviews
Maintenance	Goal: Characterize sites that sustain use of eScreening • Quant data: % of clinics and providers that sustain use (50% benchmark) • Qual data: Barriers and facilitators to maintenance from observations and interviews

*Quant* Quantitative, *Qual* Qualitative

#### Aim 3

Qualitative data from contextual elements of PRISM will be used to construct comparative analysis between high- and low-eScreening reach sites. Questions and measures assessing the PRISM contextual dimensions will be included in the qualitative interviews and observations (qualitative data) and will also be informed by the quantitative surveys and EMR data (quantitative data). Qualitative data will be analyzed as described for Aim 2, but we will use a template approach [72] for the analysis using constructs from contextual factors outlined in Table 4.

We will use codes identified and created based on the PRISM constructs and other emergent themes to tag the relevant transcript quotations. Quotation reports will list the associated quotations verbatim by the site. Sites will be divided by high vs. low reach using a cutoff score of 30% (from Aim 1), based on prior work [73]. Qualitative comparative analysis (QCA) will allow us to compare high and low eScreening reach sites to identify factors influencing the implementation of eScreening and the impact of the MCIS using systematic cross-case comparison to better understand causal complexity [74], as outlined in Table 5. A thematic analysis of site interview data will be used to supplement QCA findings.

**Table 5:** Aim 3 PRISM contextual factors for qualitative comparative analysis

PRISM construct	Proposed definitions
Program/intervention: Organizational perspective • Readiness • Addresses barriers of frontline staff • Burden (complexity & cost) • Usability and adaptability • Team sees results	• Team is ready for change • MCIS addresses barriers of staff • Burden is manageable • eScreening is usable and adaptable for the program • Team sees results
External environment • Regulatory environment	• Mandates from TCM or other VHA program office(s)
Implementation & sustainability infrastructure • Dedicated team • Adopter training and support • Relationship & communication with adopters • Adaptable protocols & procedures • Plan for sustainability	• There is an identified eScreening team • Team believes training and support were adequate • Team has positive relationships communication • Team feels protocols and procedures are adaptable • There is a plan for sustainment
Recipients: Organizational characteristics • Management support & communication • Clinical leadership • Systems & training • Staffing & incentives • Expectation of sustainability	• Management is supportive of eScreening and communicate support to frontline staff • There is clear clinical leadership • Process for training is known • Staffing is adequate • There is an expectation for sustained use

## Discussion

This stepped-wedge, mixed-method, hybrid type-II implementation trial will evaluate an innovative VHA technology, eScreening, to improve the rate and speed of suicide risk screening and referral to mental health care, as well as evaluate a strategy designed to help programs implement eScreening in new sites. The results will help to identify best practices for screening in suicide prevention for Post-9/11 veterans enrolling in VHA and provide information on how best to implement technology-based screening into clinical care programs.

Aim 1 will provide effective data for the eScreening program to improve the rate and speed of suicide risk and mental health screening in VHA TCM clinics by comparing screening with eScreening to TAU in TCM programs. We anticipate eScreening will be associated with improved rates and speed of PTSD, depression, alcohol, and suicide screening and evaluation, as well as increased referral to mental health treatment. The findings will support the use of eScreening for timely detection of, and intervention for, suicidal ideation and other mental health symptoms. Information collected from Aims 2 and 3 will provide a combination of core components of eScreening and the MCIS (including use of an adapted Lean/SixSigma RPIW), viable strategies, barriers and solutions, facilitators, promising adaptations, resources needed for implementation (including replication cost), and contextual information from each site.

These data will inform the development of an eScreening Implementation Guide that will provide step-by-step guidance and needed resources for the scale-up of eScreening across the VHA and beyond. Upon successful completion of this study, we will pursue assessing the benefit of eScreening in other programs for facilitating efficient rapid referral and measurement-based care, as well as whether and what factors predict variation in utilization of these tools across facility organization, clinic structures, and leadership support for their implementation.

This study will also contribute to expanding our understanding of how implementation science models can guide the implementation and evaluation of larger scale health delivery implementation efforts. More specifically, this study will integrate the PRISM contextual domains and RE-AIM outcomes to inform real-world delivery of eScreening across 8 VHA sites. Contextual data will be collected longitudinally (pre-implementation, implementation, sustainment) allowing for the assessment of the dynamic context across and within sites. The information provided by the longitudinal context assessment will be used real time to guide refinements for the intervention and implementation strategy. These processes are well described and recommended by Chambers and colleagues in their paper on the dynamic sustainability framework [75]. Furthermore, the use of qualitative and quantitative data from multiple sources and levels and at multiple timepoints will allow for a rich description of the complex implementation process across and within sites. The use of QCA will allow us to integrate these diverse data sources into a more coordinated understanding of what key factors contribute to the optimal implementation of eScreening. The systematic documentation of adaptations [68] and periodic reflections [69] from the research team throughout the study will generate important lessons learned for the field and guidance for future scale-up of eScreening. Overall, this trial will determine the effectiveness of an innovative screening technology while evaluating the feasibility, acceptability, and potential impact of a novel implementation strategy that will be evaluated using state-of-the-art implementation science methods. Findings will inform best practices in suicide prevention and mental health screening and will inform implementation efforts for technology.

## Data Availability

Not applicable.

## Abbreviations

AUDIT-C: Alcohol Use Disorders Identification Test
CESAMH: Center of Excellence for Stress and Mental Health
CSRE: Comprehensive Suicide Risk Evaluation
CSSRS: Columbia Suicide Severity Rating Scale
EBP: Evidence-Based Psychotherapy
EMR: Electronic medical record
HERC: Health Economics Resource Center
HLM: Hierarchical Linear Modeling
HSR&D: Health Services Research and Development Service
ICC: Intraclass correlations
IT: Information technology
MCIS: Multicomponent implementation strategy
PCPTSD: Primary Care Post-Traumatic Stress Disorder
PDSA: Plan-Do-Study-Act
PHQ-2: Patient Health Questionnaire-2
PRISM: Performance of Routine Information System Management
PTSD: Post-traumatic stress disorder
QCA: Qualitative Comparative Analysis
Qual: Qualitative
Quant: Quantitative
RE-AIM: Reach Effectiveness Adoption Implementation Maintenance
RPIW: Rapid Process Improvement Workshop
SW: Stepped-wedge
TCM: Transition Care Management
VA: Veterans affairs
VHA: Veterans Health Administration
